# Functional assays to evaluate antibody-mediated responses against *Shigella*: a review

**DOI:** 10.3389/fcimb.2023.1171213

**Published:** 2023-05-16

**Authors:** Elena Boero, Giacomo Vezzani, Francesca Micoli, Mariagrazia Pizza, Omar Rossi

**Affiliations:** ^1^ GSK Vaccines Institute for Global Health (GVGH) S.r.l., Siena, Italy; ^2^ Department of Life Sciences, Imperial College London, London, United Kingdom

**Keywords:** antibodies, functional assays, *Shigella*, serum bactericidal assay (SBA), opsonophagocytic killing assay (OPKA), invasion

## Abstract

*Shigella* is a major global pathogen and the etiological agent of shigellosis, a diarrheal disease that primarily affects low- and middle-income countries. Shigellosis is characterized by a complex, multistep pathogenesis during which bacteria use multiple invasion proteins to manipulate and invade the intestinal epithelium. Antibodies, especially against the O-antigen and some invasion proteins, play a protective role as titres against specific antigens inversely correlate with disease severity; however, the context of antibody action during pathogenesis remains to be elucidated, especially with *Shigella* being mostly an intracellular pathogen. In the absence of a correlate of protection, functional assays rebuilding salient moments of *Shigella* pathogenesis can improve our understanding of the role of protective antibodies in blocking infection and disease. *In vitro* assays are important tools to build correlates of protection. Only recently animal models to recapitulate human pathogenesis, often not in full, have been established. This review aims to discuss *in vitro* assays to evaluate the functionality of anti-*Shigella* antibodies in polyclonal sera in light of the multistep and multifaced *Shigella* infection process. Indeed, measurement of antibody level alone may limit the evaluation of full vaccine potential. Serum bactericidal assay (SBA), and other functional assays such as opsonophagocytic killing assays (OPKA), and adhesion/invasion inhibition assays (AIA), are instead physiologically relevant and may provide important information regarding the role played by these effector mechanisms in protective immunity. Ultimately, the review aims at providing scientists in the field with new points of view regarding the significance of functional assays of choice which may be more representative of immune-mediated protection mechanisms.

## Introduction

Shigellosis is a diarrhoeal disease caused by a bacterial infection of the colonic epithelium that causes great morbidity and mortality, with >200.000 global annual deaths especially in Africa and Asia (Khalil et al., 2018; Libby et al., 2023). Children younger than 5 years, in particular if not breastfed and malnourished, and adults older than 70 are at higher risk of severe disease and death (World Health Organization, 2005; Khalil et al., 2018). *Shigellae* spp. are reported to be highly invasive in humans, their only natural host; indeed ingesting as little as a few dozen of bacteria is sufficient to cause disease (DuPont et al., 1989). Transmission typically occurs *via* the faecal-oral route by exposure to infected feces, *via* person-to-person contact, ingestion of contaminated food and water, and contact with contamined fomites (Carayol and Tran Van Nhieu, 2013; Kotloff et al., 2018). In the last decades sexual contact has become an important transmission route of infection at the origin of several outbreaks in high-income countries (Kotloff et al., 2018; Charles et al., 2022). Typical treatment forsees the use of first-line and second-line antibiotic cycles (Kotloff et al., 2018), and anti-microbial resistance is rising dramatically (Organization, W.H, 2017).

The etiological agents of shigellosis are four Gram-negative *Shigella* species, *S. flexneri*, *S. sonnei*, *S. dysenteriae*, and *S. boydii*, typically classified in serotypes depending on the structure of the O-antigen (OAg), the polysaccharidic portion of the lipopolysaccharide (LPS) (Liu et al., 2008). *Shigella* shares extended genomic relatedness with *E. coli* (Lan and Reeves, 2002). During evolution, *Shigella* acquired essential virulence genes *via* an extra invasion plasmid encoding for factors enhancing its invasive capacity (Yang et al., 2005). Among these, the structural components, regulators, and effectors of the type three secretion system (T3SS) are essential for host infection, generating a needle-like structure that mediates cell entry (Yang et al., 2005).

Available seroepidemiological studies indicate that *S. flexneri* and *S. sonnei* are the most prevalent species, and according to the latest available data five *S. flexeneri* serotypes alone (2a, 6, 3a, 2b, and 1b) are responsible for more than half of all infections in low- and middle-income countries (LMICs) (Kotloff et al., 1999; Livio et al., 2014). Those, together with *S. sonnei* could count for the vast majority of *Shigella* cases worldwide (Livio et al., 2014). Anyhow, current numbers are probably underestimated, and studies are urgently needed, especially differentiated by region (Baker and The, 2018).

The important global burden of shigellosis, together with the emergence of antibiotic resistant *Shigella* prompts the urgent development of new preventive and treatment measures (Lefevre et al., 2023). *Shigella* vaccine discovery has been prioritized by the immunization, vaccines, and biologicals department of the World Health Organization (WHO), with the rationale that vaccination would decrease morbidity and mortality associated to shigellosis, as well as the use of antibiotics inducing antimicrobial resistance (AMR) (World Health Organization, 2017). Despite prolonged efforts, no licenced vaccine against shigellosis is currently available, and vaccine candidates demonstrated only partial protection against single serotypes (Mel et al., 1968; Passwell et al., 2010; Pasetti et al., 2020; MacLennan et al., 2022).

## Pathogenesis of shigellosis

*Shigella* infects the colonic epithelium, causing ulcers in the mucosa, and acute inflammation with recruitment of inflammatory cells (Mathan and Mathan, 1991; Perdomo et al., 1994; Schroeder and Hilbi, 2008; Marteyn et al., 2012; Kotloff et al., 2018). The molecular pathogenesis of shigellosis is a complex multistep process, still not fully elucidated today. *Shigella* evolved great tolerance to the extreme conditions of the gastrointestinal tract, and integrates an array of environmental cues, e.g., bile, to increase its survival and prime its invasive phenotype (Marteyn et al., 2012; Sistrunk et al., 2016; Nickerson et al., 2017; Tinevez et al., 2019; Chiang et al., 2021). To complete its infection cycle and spread in the environment, *Shigella* needs to gain access to the intracellular compartment at the colonic level. The primary route of entry of *Shigella* was identified in microfold cells (M cells), specialized cells that constitutively translocate material from the intestinal lumen to the basolateral side, to be sampled by the underlying organized gut-associated lymphoid tissue (Wassef et al., 1989; Mathan and Mathan, 1991; Perdomo et al., 1994; Sansonetti et al., 1996). *Shigella* reaching the subepithelial compartment escape macrophage phagocytosis and interacts with the basolateral surface of the epithelium, triggering its reuptake (Sansonetti et al., 1996; Cossart and Sansonetti, 2004). The idea of *Shigella* directly invading enterocytes has been traditionally neglected, since *Shigella* is not very efficient in infecting epithelia in both *in vitro* (Mounier et al., 1992) and *in vivo* models (Perdomo et al., 1994). However, a recent study using an Intestine-Chip model, which mimics the mechanical forces of shear stress and peristalsis found in the human intestine, demonstrated that low bacterial doses can significantly infect epithelial cells from the apical side. These findings challenge the idea that M cells are the only entry point for *Shigella*, and suggest current epithelial models could be oversimplified and not representative (Grassart et al., 2019).

It has been reported that adhesion of *Shigella* is regulated by environmental conditions, such as the presence of bile salts, which need to be reproduced in *in vitro* models during investigation (Faherty et al., 2012; Sistrunk et al., 2016; Chanin et al., 2019). Furthermore, the role of both classical (Chanin et al., 2019) and new adhesins has been described, such as IcsA (Brotcke Zumsteg et al., 2014), MaM (Mahmoud et al., 2016), and T3SS-secreted OspE1/OspE2 proteins (Faherty et al., 2012), but the regulation of their adhesion is T3SS-dependent and their deletion does not completely abrogate adhesion to cells (Faherty et al., 2012; Brotcke Zumsteg et al., 2014). It was proposed that *Shigella* could enhance it adhesion by binding host molecules present in the intestinal mucosa to mediate the connection to the epithelium, such as the host defence peptide human Enteric α-Defensin (HED5). Interestingly, this hypothesis could partially explain *Shigella* human host specificity (Xu et al., 2018). The adhesins required for *Shigella* attachment to the colonic epithelium may serve as ideal targets for vaccine development, but so far none of these targets has been tested in clinic as a vaccine.

Upon cell entry *Shigella* escapes the endosome and spreads to neighbouring epithelial cells, minimizing exposure to the extracellular milieu and immune cells (Wassef et al., 1989; Rey et al., 2020). During the cytosolic phase, *Shigella* replicates and spreads to adjacent epithelial cells *via* an action-based motility mechanism mediated by the outer membrane protein IcsA (or VirG) (Fukumatsu et al., 2012). Briefly, *Shigella* takes control of the host’s cytoskeleton, polymerizing actin filaments at one pole to propel itself (Bernardini et al., 1989). The push on the cell membranes forms outward pseudopodia-containing bacteria that extend into neighbouring cells. Recipient cells eventually engulf double-membraned compartments and release the bacterium in the cytosol (Fukumatsu et al., 2012).

Historically the induction of uptake by cells and endosome escape has been described to be mediated by the T3SS. The T3SS is a needle-like structure that pierces the host cell membrane and delivers effectors such as invasion plasmid antigen (Ipa) proteins in the host cell cytoplasm to modulate cell function and favour invasion. The extracellular domains of the needle and needle tip complex (Muthuramalingam et al., 2021) are involved in host-sensing and may represent important targets for antibodies. Briefly, the needle body is composed of MxiH, and a pentamer of IpaD sits on top of the needle to avoid premature translocation (Epler et al., 2012; Muthuramalingam et al., 2021). Upon environmental cues identified in bile salts and particularly deoxycholate, IpaD induces recruitment of IpaB to the needle tip complex (Olive et al., 2007; Stensrud et al., 2008; Dickenson et al., 2011; Barta et al., 2012; Bernard et al., 2017). Host cell sensing is probably mediated by IpaD and IpaB regulation, as IpaB deletion mutants lose their ability to invade, but it is not elucidated yet (High et al., 1992).

LPS mediates immune evasion, as it protects the bacterium from both extracellular and intracellular factors, such as extreme acidity of the gastric environment (Martinic et al., 2011), and protects from complement-mediated killing and phagocytosis by reducing opsonization (Lindberg et al., 1991; Fudała et al., 2003; Caboni et al., 2015). By modifying the length and structure of its LPS, *Shigella* modulates accessibility to surface proteins and thus immune sensing (Morona et al., 2003; Edwards-Jones et al., 2004; West et al., 2005; Paciello et al., 2013). The role of LPS as modulator of bacterial pathogenesis was further expanded in *S. sonnei*, which produces a polysaccharidic capsule with structural analogy to LPS OAg (Caboni et al., 2015). The presence of the capsule was found to greatly decrease the ability of *S. sonnei* to invade HeLa cells by masking relevant critical virulence proteins for invasion e.g., IpaB. On the other hand, the capsule increases resistance to complement-mediated killing and the ability to spread in a rabbit model (Caboni et al., 2015). Whether LPS has a specific role in the invasion process is not fully clarified. Studies in *in vitro* models suggest that LPS may not be necessary for cell entry as ΔOAg strains of *S. sonnei* and *S. flexneri* are routinely -used to invade HeLa and other cell types (Okamura and Nakaya, 1977). Work on polarized epithelia showed that LPS may enhance adherence and invasion *via* the basolateral membrane (Kohler et al., 2002). This is in line with the observation that mutants lacking the OAg are less virulent than their wild-type counterparts, as shown in a guinea pig model of keratoconjunctivitis (Okamura and Nakaya, 1977). Overall, the fact that naturally acquired protection against *Shigella* is serotype specific testifies the fundamental role of LPS as a fitness factor of *Shigella* (Cohen et al., 2019).

## The role of antibodies in fighting shigellosis

The use of antibody-based biologics against *Shigella* is supported by epidemiological studies showing that the natural immunity of adults and children living in *Shigella*-endemic countries protects from disease or reduces severity (Ndungo and Pasetti, 2020). Placentally-transferred IgG shields infants from infection during the first months of life (Thompson et al., 2016; Chisenga et al., 2021; Ndungo et al., 2021). High levels of *Shigella*-specific secretory IgA (sIgA) against virulence plasmid-associated antigens in maternal milk confer partial protection (reviewed in (McGuire et al., 2020)), and even predicted symptom status in a cohort of *Shigella*-infected infants (Hayani et al., 1992). Its proposed mechanism of action was investigated in a rabbit ileal loop infection model and was attributed to the ability of the antibodies to reduce the invasion (“immune exclusion”) of the colonic epithelium and to the downregulation of the detrimental inflammatory response (Boullier et al., 2009).

LPS, the most abundant surface antigen of *Shigella*, has been identified as potential protective antigen during epidemiological as well as clinical studies (Cohen et al., 2019). Evidence has been accumulated that serum IgG antibodies against *Shigella* LPS can be a mechanistic immunological correlate of protection (Plotkin, 2020). Recently a re-analyses of serologic and vaccine efficacy data from two trials with *Shigella sonnei* glycoconjugate in adults and children aged 1–4 years in Israel confirmed the association between anti–*S. sonnei* LPS antibodies with vaccine efficacy (Cohen et al., 2023).

Subunit vaccine candidates (extensively reviewed in (MacLennan et al., 2022)) are based on different technologies to display OAg to the immune system, ranging from classical conjugates (Taylor et al., 1993), to synthetic carbohydrates conjugated to carrier protein TT (Cohen et al., 2021), to bioconjugates to recombinant exotoxin A of *Pseudomonas aeruginosa* (rEPA) (Clarkson et al., 2021; Martin and Alaimo, 2022), to delivery system represented by outer membrane vesicles (called Generalized Modules for Membrane Antigens - GMMA) purified from genetically modified *Shigella* (Micoli et al., 2022). Unfortunately, because of *Shigella* OAg hypervariability, a combination of OAg from different serotypes is needed to induce broad protection against shigellosis, despite a certain level of cross-reactivity among *S. flexneri* OAg, which could result in cross-protection (Citiulo et al., 2021). Antibodies against *Shigella* proteins, including virulence factors such as Ipa, are also present in the serum of infected subjects (Oaks et al., 1986; Pasetti et al., 2020). Compared to hypervariable LPS, protein antigens present the advantage of being more conserved across *Shigella* spp. High anti-T3SS protein titres were found in cord blood of infants and naturally infected people, suggesting a protective role (Ndungo et al., 2018; Pasetti et al., 2020; Ndungo et al., 2021). Vaccine approaches targeting *Shigella* proteins have been attempted e.g., against outer membrane protease IcsP (Kim et al., 2015), however in vaccines currently in clinical development protein antigens are combined with LPS (e.g., containing IpaB and IpaD proteins complexed with *S. flexneri* 2a LPS (Turbyfill et al., 2022)).

## Serological assays for *Shigella*

This review is focused on the dissection of the systemic humoral response against *Shigella* by the use of serological assays to quantify antigen-specific antibody levels and their functionality. Beyond quantification of antibodies, functional assays are serological methods that enable to measure *in vitro* the ability of antibodies to neutralize or inhibit key effector functions of the pathogenicity of *Shigella*. The dissection of mucosal humoral response is out of the scope of this review, nonetheless, some serological assays described below (e.g., binding assays, adhesion/invasion inhibition assay) could be applied to secretions of the gastrointestinal tract, such as feces or rectal swabs, to provide information about mucosal antibodies.

The most widely used assay to determine the specificity of antibody binding to single antigens and quantify their abundance in serum or other samples is represented by the enzyme linked immunosorbent assay (ELISA). Briefly, antigen-coated plates are incubated with the sera of interest and antibody binding is revealed though binding of a secondary antibody directed against the host species of the primary antibody, conjugated with enzymes (e.g., alkaline phosphatase) to amplify the signal with the substrate of interest. The ELISA is very simple, can be automated to increase throughput and operator independency, and can be easily transferred to different labs including those with low level of technology. Flow cytometry is instead commonly used to assess serum binding to whole *Shigella* instead of single antigens (Mancini et al., 2023).

Multiplex technologies (e.g., Luminex or MesoScale) offer the extra option to interrogate up to dozens of antigens simultaneously, enabling the operator to investigate quantity, specificity, and cross-reactivity of the serum against several antigens in parallel. Throughput wise, ELISA and Luminex assays can be developed in 384-well plates format, granting a high throughput with low quantity of sample needed to perform the assay. Considering the high number of *Shigella* OAg types, multiplexed immunoassays provide the most convenient and sample-saving method to screen for multiple OAg in naturally infected as well as vaccinated individuals. A 6-plex panel containing IpaB, IpaC, IpaD and purified LPS from *S. sonnei*, *S. flexneri* 2a, and *S. dysenteriae* has been published where Luminex titers were confirmed to correlate with ELISA (Kaminski et al., 2013).

Antigen expression/extraction, purification, and modification for coating or coupling purposes inevitably introduce changes compared to natural antigens. Among the different key antigens important for shigellosis pathogenesis, LPS (containing the serotypical OAg) purified by bacteria using hot phenol extraction was chosen as a consensus coating antigen in immunological assays to determine antibody titres against different serotypes of *Shigella* (Hayani et al., 1992).

Additional analyses better define the class/subclasses profile and the affinity/avidity of the antibodies. With antibodies being complex molecules, functionality results from the contribution of Fab-related attributes (e.g., targeted epitope, affinity, and avidity) as well as Fc-mediated effector functions (e.g., interaction with immune cell receptors, host cell-signalling, antigen presentation, initiation of the complement cascade). Therefore, functional assays should be integrated with assays designed to characterize and dissect above-mentioned relevant attributes of the immune response. Antibody subclasses in particular could predict at least in part antibody functionality, as the effector function is regulated at the level of the tail region of immune-complexed antibodies by protein differences and glycosylation (Wang and Ravetch, 2019). Experts in the field agree that the humoral response deserves a more in-depth characterization through the use, among others, of functional assays, usually serum bactericidal assay (SBA) or opsonophagocytic killing assay (OPKA) (Kaminski et al., 2019).

## Functional assays in shigellosis

To evaluate the protection induced by vaccine formulations following active and/or passive immunization at the preclinical level, challenge models in animals are often used. Since *Shigella* is a human-restricted pathogen, animal infection models currently developed to assess the potential protective efficacy of candidate vaccines do not mirror well human infections (Lederer et al., 2005; Brunner et al., 2019). Proposed small animal challenges include guinea pig model (intrarectal route) (Shim et al., 2007), keratoconjunctivitis in guinea pigs, rabbits, or mice (Sereny test) (Sereny, 1957), rabbit ligated ileal loop model (injection) (Sansonetti et al., 1983; Wassef et al., 1989; West et al., 2005; Medeiros et al., 2020), mouse pulmonary model (intranasal route) (Mallett et al., 1993), and mouse peritoneal model (Yang et al., 2014). The group of R. Guerrant developed a murine model of diarrhoea that allowed the oral administration of bacteria, but with a considerable inoculum of 10^8^ bacteria per mouse (PH et al., 2019; Medeiros et al., 2020). Finally, Mitchell and colleagues developed an oral infection mouse model in NAIP–NLRC4 inflammasome deficient mouse that become susceptible to shigellosis, however infection is only possible prior to clearance of the microbial flora *via* streptomycin treatment, possibly altering the first steps of the invasion process (Mitchell et al., 2020). Eventually, testing candidate *Shigella* vaccines in humans seems the most valuable way to assess their efficacy, by controlled human infection models (CHIMs) (Frenck et al., 2020; Frenck et al., 2021; Talaat et al., 2021). However, CHIMs are usually conducted on naïve, adult subjects rather than in infants in LMICs, thus their immune reponse might not be representative of the target population. Upon identifying a protective immune response, this must be dissected not only qualitatively, but also functionally to identify surrogates of protection. This evaluation must necessarily rely on functional assays (McArthur et al., 2017).

*In vitro* assays evaluating antibody functionality are key to better characterize the features of the elicited humoral response, aiding vaccine development. In contrast to methodologies evaluating antibody quantity, or simply binding to bacteria like flow cytometry, functional assays mimic the ability of the host immune response to counteract infection mechanisms. Indeed, different effector functions of antibodies might act in specific niches of the host or certain steps of the lifecycle of the infection, with a pathogen that can modulate the expression of antigens as a mechanism to infect and in turn evade the host response.

**Figure 1:**
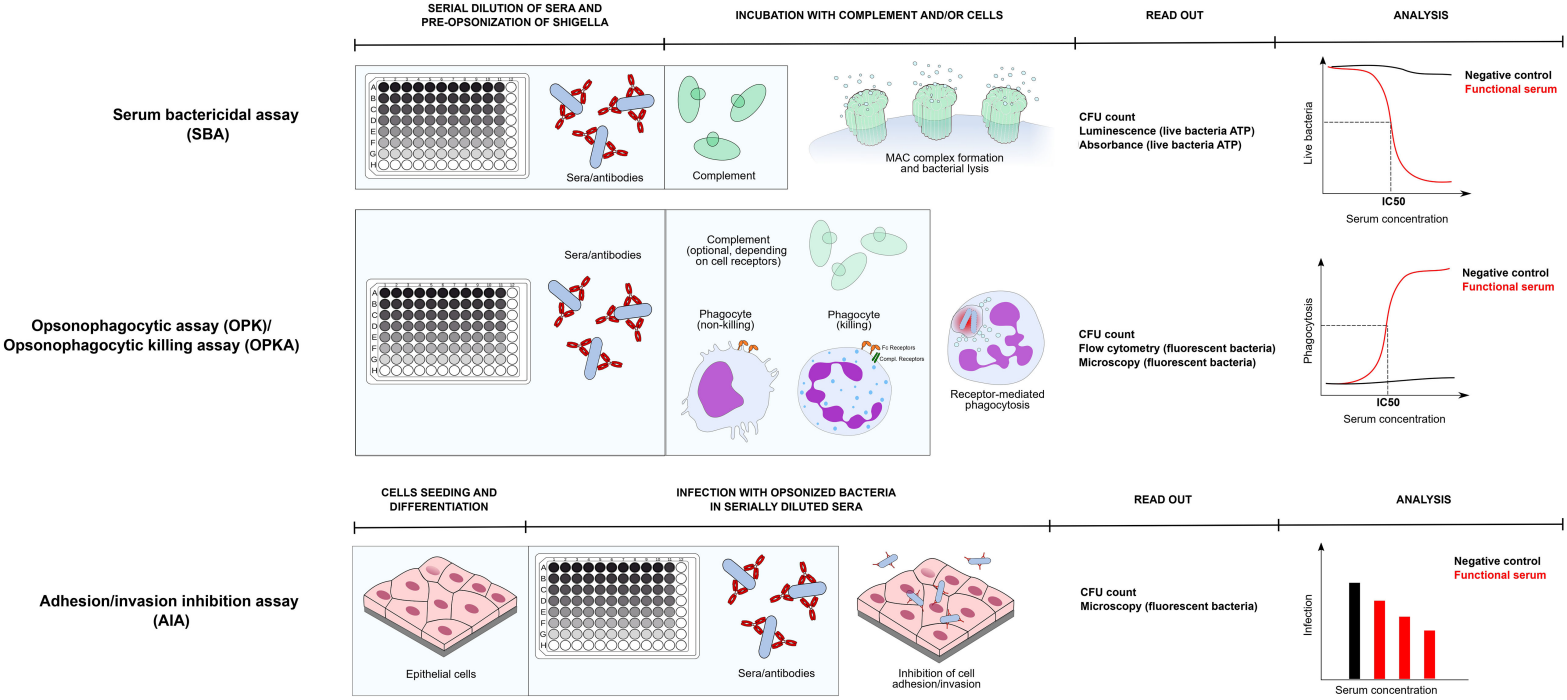
Assay protocol of three main functional assays to dissect antibody responses against *Shigella*: SBA, OPKA, AIA. Protocols of each assay is represented in its main steps. All assays are assumed to be prepared in a 96 well format. *Serum Bactericidal Assay* - During SBA and OPA/OPKA sera are first serially diluted in the plate and incubated with bacteria (preopsonization phase). Subsequently an exogenous complement source is added and incubated for a time ranging from 15 minutes to 3 hours. During SBA bacterial lysis occurs, which can be verified by means of CFU count or by quantifying ATP of live bacteria *via* luminometric or colorimetric methods. Bactericidal activity of serum is represented as the quantification live bacteria plotted against serum concentration and the 50% inhibitory concentration is calculated. *Opsonophagocytosis assay/Opsonophagocytic killing assay* - In OPA/OPKA opsonized bacteria with serially diluted sera are incubated together with phagocytic cells. Depending on the phagocytic receptors profile of the phagocyte (Fc receptors + complement receptors), complement may or may not be included. Depending on the cell type and its differentiation stage, phagocytes only uptake bacteria, or also kill them in the phagolysosome. Uptake and clearance of bacteria are commonly monitored by CFU count, usually upon cell lysis, or by monitoring of engulfed fluorescent bacteria by flow cytometry and/or fluorescence miscroscopy. Quantification of phagocytosis is plotted against serum concentration and similarly to SBA an IC50 value is extrapolated. *Adhesion/invasion inhibition assay* – the epithelial cell model of choice is prepared, possibly in a 96 well plate format. Subsequently, preopsonized bacteria are added to the cells for infection and excess bacteria are removed by washing. Adhesion/invasion are evaluated by CFU count or by fluorescence microscopy and values are usually represented as the number of infecting bacteria or percentage of infecting bacteria compared to negative control against serum concentration.

**Figure 2:**
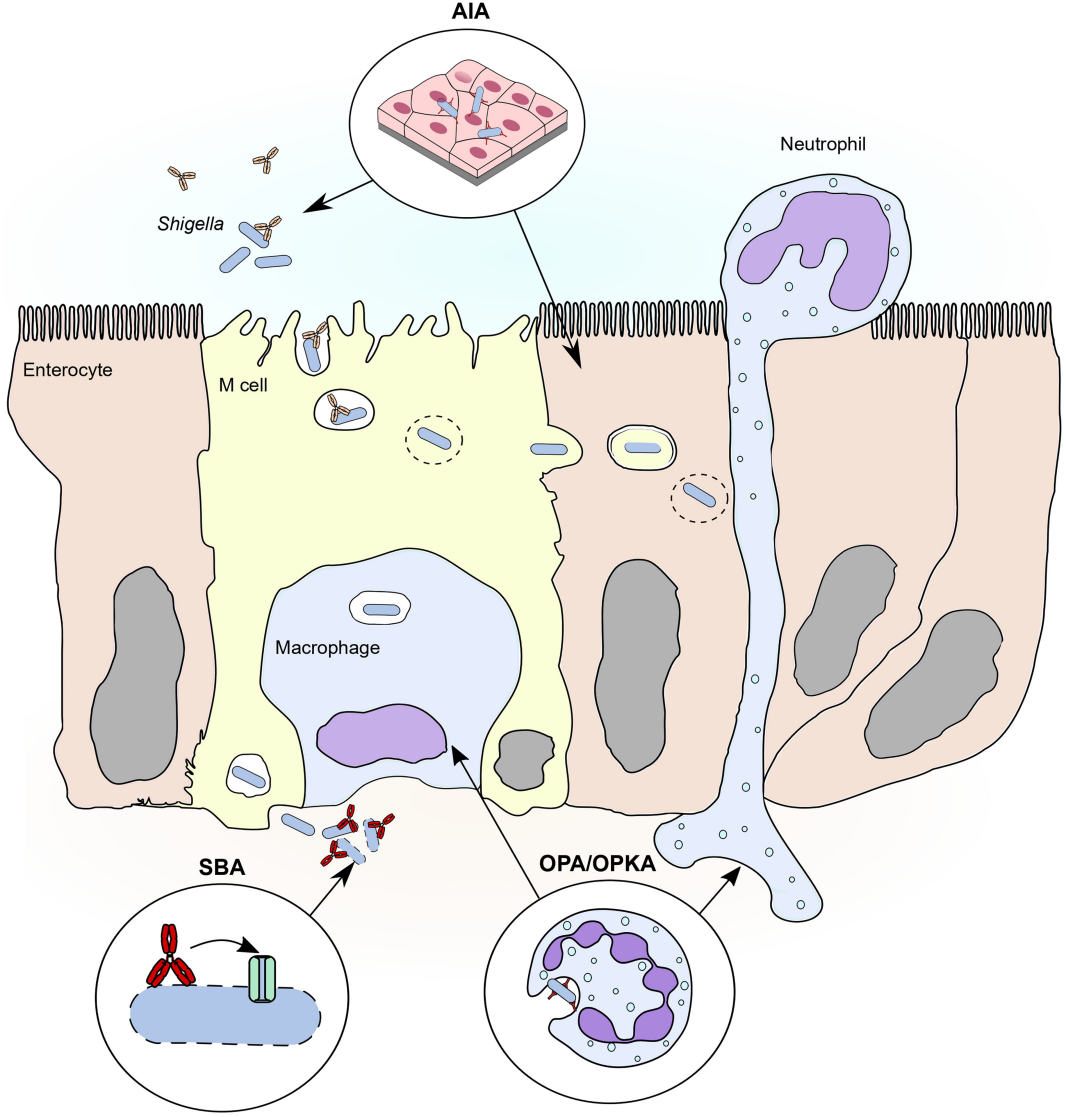
Major functional assays to evaluate humoral response against shigellosis in relation to steps of the pathogenesis. Three main functional assay approximate three important steps of the pathogenesis of shigellosis. 1) *Adhesion/invasion inhibition assay (AIA)*. The adhesion/invasion inhibition assay reproduces the first phase of infection, in which *Shigella* approaches the colonic epithelium from the intestinal lumen, possibly *via* engulfed by M cell endosomes. Adhesion assays evaluate the role of mucosal antibodies in neutralizing relevant invasion factors that mediate contact of *Shigella* with M cells and/or epithelial cells. Invasion cells also evaluate the role of antibodies in inhibiting endosome escape and the spreading of *Shigella* from cell to cell in the epithelium. 2) *Serum bactericidal assay (SBA)*. Serum bactericidal assay reproduces the conditions in which *Shigella*, once reached the basolateral side of the epithelium, is opsonized by complement proteins. Complement opsonins deposit on the membrane forming a pore that eventually lyses the bacterium, possibly controlling spreading and reinfection from the basolateral side. Antibodies enhance compement deposition, thus enhancing complement-mediated lysis of *Shigella* and their contribution can be investigated by serum bactericidal assay. 3) *Opsonophagocytic killing assay (OPKA)*. During infection *Shigella* interacts with two major phagocytic cell types: neutrophils, recalled to the site of infection from the from the circulation, and resident macrophages. Opsonophagocytic killing assays approximate the interaction between phagocytes and *Shigella* and predict the role of antibodies in inducing efficient uptake and clearance of the bacteria.

Functional assays can be also aimed at measuring antibody-mediated immunological effector functions, such as phagocyte-mediated bacterial killing or complement-mediated lysis. The correlation between the results of these functional assays and favourable clinical outcomes in *Shigella* CHIMs or clinical trials has been reviewed extensively by Ndungo & Pasetti (Ndungo and Pasetti, 2020) and by the group of G. Alter (Bernshtein et al., 2022). Here we present an overview of the functional assays used in *Shigella* vaccine development. To facilitate the reading, in **Figure 1** we report a simplified protocol for each main group of functional assays described below: serum bactericidal assay (SBA), opsonophagocytic killing assay (OPKA), and adhesion/invasion inhibition assay (AIA). In **Figure 2** we represent the physiological relevance of each functional assay.

## Serum bactericidal assays

Serum bactericidal assays (SBA) evaluate the functionality of opsonizing antibodies by measuring complement-mediated lysis of Gram-negative bacteria such as *Shigella* spp. Antibodies of relevant subclasses binding the bacterial surface spark the classical and alternative pathways of complement activation, depositing complement elements that culminate into the assembly of a lytic pore (BOX1). SBA is the most widely functional assay used to measure the functionality of antibodies against Gram-negative bacteria by vaccine developers (Holst et al., 2003; Borrow et al., 2005; Plotkin and Gilbert, 2012; Boyd et al., 2014; Rossi et al., 2020; Micoli et al., 2021). Indeed, SBA is routinely used to dissect the humoral response against *Shigella* spp., either from naturally exposed individuals, challenged, or vaccinated patients (Kaminski et al., 2019; Bernshtein et al., 2022; Kapulu et al., 2022). High SBA titres have been associated with some degree of protection from moderate to severe disease in several studies, suggesting that SBA may be a functional correlate of protection recapitulating a positive role of antibodies during the pathogenesis of *Shigella* (Shimanovich et al., 2017; Clarkson et al., 2020). Bactericidal activity is mainly attributed to anti-LPS/OAg antibodies, probably since LPS provides abundant and repetitive surface antigens that favour antibody hexamerization and thus complement deposition (Rossi et al., 2020; Mancini et al., 2022).

The role of bactericidal activity *per se* in the context of the pathogenesis of shigellosis remains to be elucidated as the infection usually remains localized in the colonic mucosa and extra-intestinal complications are rare (Kotloff et al., 2018). Bacterial lysis can only occur beneath the epithelium where the extracellular matrix is relatively rich in complement and serum antibodies. SBA titres could therefore provide a useful indication of how protective antibodies could enhance *Shigella* complement sensitivity and ability to prevent reinfection from the basolateral side. The intestinal lumen could also be a disregarded setting of complement-mediated lysis since IgM and IgG and at least a part of complement elements was found on the intestinal surface (Sina et al., 2018; Chen et al., 2020). How SBA titres could reflect mucosal immunity in this regard is a matter of investigation.

The assay protocol forsees that bacteria are incubated with a source of opsonizing antibodies of interest, together with an exogenous complement source, most commonly baby rabbit complement (BRC) (**Figure 1**). Antibodies to be tested activate complement, leading to the lysis of bacteria. The outcome of the assay is usually represented as a 50% inhibitory concentration (IC50), which indicates the concentration of antibodies or serum required to kill half of total bacteria compared to the negative control. Several improvements to the classical assay have been achieved, mostly aimed at decreasing the duration of the assay and enhancing its throughput, including the miniaturization of the assay to a 384-well format (Aruta et al., 2021) and the simplification of readouts compared to plating and counting surviving bacterial colony forming units (CFU) (Nahm et al., 2018). Alternative methods based on colorimetric, luminometric and/or fluorometric readouts have converted SBA to a one-day assay. These readouts are usually derived from reactions with metabolic reagents by the live bacteria. Necchi et al. developed a luminescence-based SBA (L-SBA) protocol which detects adenosine triphosphate (ATP) production by live bacteria (Necchi et al., 2018) and the assay has been optimised and characterised (Rossi et al., 2020; Mancini et al., 2022) to analyse clinical samples from multiple studies (Frenck et al., 2021; Micoli et al., 2021; Kapulu et al., 2022). Resazurin (also known as alamarBlue) offers an alternative colorimetric and fluorometric endpoint (Romero-Steiner et al., 2004). Limitations of the assay include the fact that assay conditions need to be adjusted to each *Shigella* strain used depending on its intrinsic complement sensitivity, i.e., how much bacteria are lysed by spontaneous complement activation.

### Box 1: classical pathway of complement activation

Complement activation culminates with the formation of pores on bacterial membranes called membrane attack complexes (MAC), ultimately lysing, and killing sensitive bacteria (Doorduijn et al., 2019).

IgM and IgG antibodies bound to epitopes on bacterial surfaces can spark local complement deposition *via* the classical pathway (Strasser et al., 2019; Chen et al., 2020). The cooperation of surface-bound antibodies is a prerequisite for efficient complement activation. Indeed, while IgM is already in pentameric form, pentamerization/hexamerization of IgGs determines conformational changes of their Fc portions that allow the docking of the C1 complement complex (Diebolder et al., 2014). C1 complexes comprises serine proteases C1r and C1s that start the enzymatic cascade of complement humoral proteins (Gaboriaud et al., 2014; Mortensen et al., 2017). C1s cleaves C4 into soluble C4a and the complement opsonin C4b. Surface-bound C4b-C1s_2_ complex cleaves C2 into C2a and C2b. C2a becomes incorporated in a complex C4bC2a, referred to as the C3 convertase, which cleaves C3 into surface-bound C3b, the central protein of the complement cascade and a phagocytic opsonin. C3b deposition quickly self-amplifies *via* the alternative pathway of complement activation, gaining thousands of C3b molecules on the bacterial surface in minutes. C3b and its cleavage product iC3b interact with phagocytic receptors on phagocytic cells to mediate internalization and killing. C3b interacts with both C3 convertases to form the C5 convertases (C4b2a3b/C3bBb3b), which convert soluble C5 into the anaphylatoxin C5a and C5b, the first component of the MAC complex (Heesterbeek et al., 2019; Doorduijn et al., 2020). C5b quickly interacts with C6 and then C7, the first proteins spanning the lipid bilayer. C5b-7 then recruits C8 and 18 copies of C9 forming the MAC complex, and is assembled from five complement proteins (C5b, C6, C7, C8 and multiple C9 proteins) (Heesterbeek et al., 2019; Doorduijn et al., 2020).

## Phagocytosis and killing assays (OPA/OPK)

Opsonophagocytosis assays (OPA) and opsonophagocytic killing assays (OPKA) measure the ability of antibodies to mediate the internalization and/or killing of *Shigella* by phagocytes. Phagocytosis assays of *Shigella* not only offer a way to measure the ability of antibodies to engage effector immune cells but could provide clues on the pathogenesis of the disease. During infection, *Shigella* interacts with both resident subepithelial macrophages underneath Peyer’s patches and recruited polymorphonuclear cells (PMNs). Both cell types sense the opsonized bacteria *via* phagocytic receptors - Fcγ receptors (FcγR) and complement receptors (CR1 and CR3) - and eventually phagocytose and clear the pathogen (Perdomo et al., 1994; Sansonetti et al., 1996; Sansonetti, 2001; Schroeder and Hilbi, 2008). However, phagocytosis may represent a double-edged sword, as excessive inflammation amplified by phagocytic cells may have a negative outcome for the host. Indeed, *Shigella* manipulates the inflammatory response inducing pyroptosis of subepithelial macrophages with the release of tissue-damaging pro-inflammatory cytokines (Clerc et al., 1987; Sansonetti, 2001; Ashida et al., 2021) and necrosis of PMNs with the release of toxic granule contents, contributing to the destruction of the epithelial barrier (Francois et al., 2000). Furthermore, it was proposed that PMNs recruited to the site of infection to the lamina propria could transmigrate through the epithelial layer offering an additional route of entry as well as a destructive inflammatory milieu. The suppression of PMNs recruitment, together with the dampening of the associated pro-inflammatory cytokines *in vivo*, preserved epithelial integrity (Perdomo et al., 1994). These aspects of the pathogenesis of shigellosis encourage setting up and using phagocytosis assays to verify not only the uptake, but also the prompt and effective clearance of *Shigella*, and whether the presence of antibodies can modulate the outcome in favour of clearance and contain collateral damage.

In a phagocytosis assay protocol, phagocytic cells are incubated with opsonized *Shigella* for the appropriate time. Internalization and/or killing of bacteria (or surrogates of bacteria) are then evaluated *via* multiple possible readouts, including CFU counting of colonies (Shimanovich et al., 2017; Ndungo et al., 2021), or flow cytometry-based (Bernshtein et al., 2022) (**Figure 1**). Depending on the combination of cell types and opsonins used, the assay provides different indications. Non-differentiated cells like THP-1 monocytes are not able to kill and only provide indication of antibody-mediated internalization of targets. In contrast, by using more specialized cells (e.g., primary neutrophils, differentiated HL-60, differentiated THP-1 macrophages) it is possible to evaluate killing efficiency of serum (Butler et al., 2019; Mancini et al., 2023). Complement is commonly added to the assay to evaluate concerted FcγRs- and CR-mediated uptake and killing (although it is not always necessary to induce internalization and/or killing) (Mancini et al., 2023).

OPKA were already used to evaluate the functionality of clinical samples. A classical OPKA with differentiated HL-60 was first established to evaluate killing of *Shigella* opsonized with serum from vaccinated and challenged patients in the presence of BRC, to eventually correlate functional antibody activity and protection (Shimanovich et al., 2017). The same assay was applied to measure the functional response induced by protective maternal blood compared to the cord blood of the corresponding baby (Ndungo et al., 2021). Both OPA and OPKA were inserted in the system serology panel of functional assays, with the difference that OPKA was performed with primary human neutrophils instead of differentiated HL-60. Flow cytometry-based OPA with THP-1 monocytes and neutrophils from whole blood were used to evaluate opsonophagocytosis of beads coated with antigens of interest, and not whole *Shigella* (Bernshtein et al., 2022) Interestingly, while SBA and OPKA results usually correlated with each other and with respect to symptoms severity, OPA assays with specific antigens provided new insights, as contrarily to SBA and OPKA titers, OAg- and IpaB-antibodies negatively correlated with symptoms severity (Bernshtein et al., 2022). Finally, classical OPKA was used by our group to evaluate the immune response of mice immunized with candidate GMMA-based vaccine (Mancini et al., 2023).

OPA and OPKA present several difficulties, among which the tuning of complement and cell conditions, which must be finely adjusted to appreciate phagocytosis-derived killing and not serum bactericidal assay. Furthermore, when flow cytometry or fluorescence microscopy are used to acquire the experiment conditions to achieve sufficient fluorescent of each bacterial strain must be optimized.

## Adhesion/invasion inhibition assays

Invasion assays have been extensively used to identify targets mediating host-pathogen interaction and define the invasion of host cells mainly through T3SS (Skoudy et al., 2000). IpaB was described to interact with HeLa surface receptor CD44 and cholesterol in the lipid rafts, while the IpaB-CD complex binds to integrin α5β1, anchoring the bacterium on the cell surface and facilitating the creation of the pore on by the T3SS (Yang et al., 2015).

Adhesion/invasion inhibition assays (AIA) were used to demonstrate the ability of antibodies, especially against Ipa proteins, to reduce virulence. Generalizing the assay protocol, *in vitro* cell models of variable complexity (discussed below), usually organized in a 96-well plate format, are infected with a defined number of live bacteria opsonized in solution with the antibodies of interest (**Figure 1**). Incubation time is adjusted depending on the desired readout: a few minutes to better appreciate adhesion, to a few hours to better appreciate invasion. Usually, excess bacteria are then rinsed off the cells by repeated washing steps. Finally, adhesion/invasion rates of bacteria can be evaluated against non-opsonized controls by two main methods: either CFU count of total or internal bacteria (upon gentamycin treatment of cells to kill external bacteria), or by immunofluorescent microscopy, possibly with a counterstain to distinguish internalized and external bacteria.

Animal anti-sera and monoclonal antibodies against IpaD (Sierocki et al., 2021), IpaB (Mills et al., 1988; Shen et al., 2010; Li et al., 2022) and IpaC (Mills et al., 1988) were demonstrated to reduce the ability of virulent bacteria to infect in *in vitro* cell models. These works are in line with studies in animal models in which mice immunized with IpaB and IpaD developed a protective immune response (both IgG and IgA) upon challenge (Martinez-Becerra et al., 2012), therefore demonstrating *in vivo* the relevance of anti-Ipa antibodies. However, neutralization of Ipa proteins could be impaired by factors such as environmental cues influencing translocation from the cytosol to the bacterial surface, transient accessibility of Ipa in the tip complex to antibody binding (Skoudy et al., 2000).

In addition, invasion assays were used to assess the importance of OAg, not only in the context of SBA, but during the infection in general. Indeed, Chowers and colleagues showed that sera from children immunized with *S. flexneri* 2a or *S. sonnei* OAg fraction of LPS inhibit the invasion of the homologous strain on the epithelial cells Caco-2 (Chowers et al., 2007). Caboni et al. also demonstrated that the presence of the OAg-homologous capsule of *S. sonnei* impairs invasion of HeLa cells (Caboni et al., 2015).

More complex models of differentiated epithelia include the co-culture of Caco-2 and RajiB cells (a human lymphoid cell line), to produce a mature intestinal follicle-associated epithelium with transcytosing M cells (Beloqui et al., 2017; Rey et al., 2020). This model was used to propose the M cell-to-enterocyte pathway as the major dissemination pathway for *Shigella* to avoid the basolateral compartment (Rey et al., 2020). 2D human intestinal enteroids (HIE) from primary tissue were used as an infection model, confirming preferential infection from M cells and the basolateral compartment (Koestler et al., 2019; Llanos-Chea et al., 2019; Ranganathan et al., 2019). HIE can be co-cultured with PMNs to study the interplay of *Shigella* with respect to PMN-epithelial cell interaction (Lemme-Dumit et al., 2022). Finally, 3D epithelial organoids derived from primary human tissue could be an interesting model for *Shigella* infection, especially apical-out organoids with reversed polarity would represent another useful model to investigate invasion on the apical vs. the basolateral side of the epithelium (Co et al., 2021).

As already mentioned, *Shigella* was traditionally observed to have a scarce ability to invade polarized *in vitro* cell models from the apical surface (Mounier et al., 1992), so much so that in many experimental models bacteria must be forced on cells by centrifugation, and *Shigella* mutants that express heterologous proteins to artificially enhance adhesion are used (e.g., AfaE adhesin (Skoudy et al., 2000), AfaI adhesin (Rey et al., 2020)), even in the presence of M cells. Whether the adhesion/invasion mechanism of *Shigella* in these cultures is faithful to the physiology of infection is a matter of debate.

The state of immunoglobulins in the intestinal lumen remains a fundamental topic to determine the ratio of function/non-functional mAbs. One example is given by the fact that mAbs are not found only as single entities in the luminal tract of the intestine, but also in complex with the secretory component (SC) (Cerutti and Rescigno, 2008). The SC is the soluble part of the polymeric Ig receptor, which is responsible for the transmigration of the IgG from the basolateral to the luminal space of the intestinal tracts (Turula and Wobus, 2018). When the IgG-polymeric Ig receptor complex is exposed in the luminal cavity, the proteases present in the digestive tract cleaves the transmembrane component of this receptor from the SC, which remains attached to the IgGs (Wei and Wang, 2021). The SC-IgG complex favors both the half-life of IgG in the luminal tract of the intestine and the effector functions of the IgG itself (Stadtmueller et al., 2016). Indeed, it was shown that only in the presence of SC an LPS-specific monoclonal antibody against *Shigella flexneri* exerts its protective role on epithelial cells (Mathias et al., 2013).

### Contact haemolysis assay

T3SS-mediated membrane lysis, possibly by translocon-induced pore formation (Chen et al., 2021), requires contact with eukaryotic cell surface. Exploiting this property, Sansonetti and colleagues set up a contact haemolysis assay in which *Shigella* is incubated with sheep erythrocytes, causing the release of haemoglobin in solution, and thus providing a colorimetric readout for membrane lysis (Clerc et al., 1985; Sansonetti et al., 1986). Haemolysis was demonstrated to be T3SS-dependent and to be 1) temperature-dependent, since the expression of the T3SS is known to occur at 37°C and not at 30°C; 2) plasmid-encoded, it did not occur when non-invasive *Shigella* spp. were tested; 3) contact-mediated, adhesion between bacteria and erythrocytes must be induced by pelleting of bacteria on red blood cells.

Interestingly, Clerc et al. demonstrated that the amount of contact haemolysis of *Shigella* spp. was correlated to the ability to penetrate HeLa cells, suggesting that the contact haemolysis assay might represent a time- and cost-effective approximation of the initial steps of the lysis of the phagocytic vacuole, a key step for intracellular multiplication of the bacterium in the colonic epithelium (Clerc et al., 1985).

Since 1986, contact haemolysis assay was reprised in subsequent works to demonstrate membrane disrupting activity of virulence factors and study the role of T3SS translocons IpaBCD. To this date, only Sieroki et al. used contact haemolysis as a functional assay to demonstrate the ability of cross-reactive anti-IpaD (*Shigella*) -SipD (*Salmonella*) monoclonal antibodies to inhibit membrane lysis (Sierocki et al., 2021). Interestingly, the tested antibody (mAb IpaD-318) was found to enhance the haemolysis of erythrocytes, but not HeLa cell invasion. The authors hypothesized that mAb IpaD-318 stabilized polymerized IpaD in an intermediate conformation that lost the capacity to control correct IpaB and IpaC translocation (Sierocki et al., 2021). Antibody fragments against IpaD were also reported to significantly reduce erythrocytes haemolysis by wild-type *S. flexneri* (Barta et al., 2017).

## Systems serology approach

No single antibody specificities, qualities, or effector functions investigated so far seem to be absolute predictors of protective immunity in shigellosis; it is thus possible that extra antibody functions and/or the integration of different aspects predict a successful humoral immunity against *Shigella*. The system serology approach, first developed by the group of Galit Alter in 2015, interrogates clinical serum samples *via* an array of different serological assays, and by means of machine learning and other high dimensional tools it then integrates the results to define the specific antibody profiles that correlate with favorable patient outcomes in various diseases, including shigellosis (Chung et al., 2015; Arnold and Chung, 2018). The profiling interrogates Fc-effector functions *via* functional assays and also forsees biophysical measurements (such as binding to Fcγ receptors and binding to specific antigens) (Chung et al., 2015).

Bernshtein and colleagues applied the systems serology approach to profile the sera of patients challenged with *S. flexneri 2a* in a CHIM (Bernshtein et al., 2022). In their study they employed the following high throughput quantitative antibody assays (Bernshtein et al., 2022): *via* Luminex they determined the subclass or isotype (IgG1-2-3, IgA1-2, IgM) of antibodies binding to antigens of interest (multiple LPS types, IpaD, IpaB, IpaC, IpaH, VirG). Interestingly, Fcγ receptor binding to immune-complexed antigens was strongly recommended as a possible predictor of antibody effector function, an aspect that is not routinely used in serological labs (Arnold and Chung, 2018; Bernshtein et al., 2022).

Functional assays (Complement deposition, SBA and OPA/OPK) were performed both on opsonized beads and on live *Shigella*. To measure complement deposition, immune complexes were incubated with baby rabbit complement to determine the ability of antibodies to start the complement cascade. To our best knowledge, this assay is also not routinely used, especially on protein targets since complement deposition on live *Shigella* is primarily driven by anti-LPS antibodies. However, this assay could help recapitulate useful functional aspects of antibodies with protein specificity, for example revealing that some protein targets could still be drivers of complement deposition when they are more exposed on the bacterial surface. Complement deposition on beads is combined with classical SBA on live *Shigella*.

At least three main and complementary functional assays were described phagocytosis: antibody-dependent neutrophil phagocytosis (ADNP) in whole blood, antibody-dependent cellular (monocyte) phagocytosis (ADCP), both performed with immune-complexed beads, and classical OPK with live *Shigella* (Butler et al., 2019; Karsten et al., 2019; Bernshtein et al., 2022).

## Discussion

Vaccines targeting human pathogens are fundamental tools for improving global health and combating emerging antimicrobial resistance. *In vitro* assays evaluating antibody biophysical properties and antibody functionality are key to identifying the features of a protective humoral response, greatly aiding vaccine development. In absence of a clear indication regarding the classes or subclasses of antibodies that are relevant for protection (e.g., IgA vs. IgG) and which is the critical and validated level that correlates with clinical protection for different age groups, it is key to prove ability of antibodies to neutralise, and better to kill, the activity of a pathogen. However, functional assays are pathogen-specific and recapitulate only precise steps of the host pathogen interactions. From the technical point of view a functional assay needs to offer sufficient throughput to allow screening of multiple samples in a cost-effective manner. Therefore, the ideal functional assay balances physiological relevance, suitable throughput, and cost-effectiveness.

In this review we presented the main functional assays used to evaluate the functionality of anti-*Shigella* antibodies, and we discussed that each assay could approximate different stages of *Shigella* mechanism of infection (**Figure 2**). SBA titres have been proven to correlate with increased protection linked to efficient antibody-induced complement mediated killing. However, we could speculate that the valence of SBA could extend beyond bacterial lysis, indirectly providing a general indication on the efficacy of opsonization on live bacteria, which is at the heart of multiple immune defence mechanisms. It is well known that SBA titers speak with OPA/OPK titers (West et al., 2005; Brotcke Zumsteg et al., 2014), since the two mechanisms rely on antibody-dependent complement deposition which can signal to phagocytes *via* Fcγ receptors and phagocytic complement receptors CR1 and CR3. Complement deposition on bacteria also makes an important intracellularly defence signal that favour autophagy and clearance of intracellular pathogens (Wang and Ravetch, 2019), and SBA titers could also reflect that.

The penetration of *Shigella* in the colonic epithelium is arguably the most crucial step in the pathogenesis of shigellosis to be inhibited by antibodies. For this reason, functional assays measuring the capacity of antibodies to prevent adhesion and/or invasion of *in vitro* epithelial models are fundamental tools. Importantly, adhesion/invasion assays are the only assays simulating events occurring at the level of the intestinal lumen, opening interesting options in testing the significance of mucosal subclasses of antibodies. Despite the clear importance, no adhesion/invasion assays were implemented in clinical testing so far.

Among the difficulties in setting up such functional assay, there is identifying a relevant target/s of the antibodies to be tested. In fact, effector functions of antibodies might act in specific niches of the host or in certain steps of the lifecycle of the infection, while the pathogen can modulate the expression of antigens as mechanism to infect and in turn evade the host response. While SBA was shown to be mainly driven by antibodies targeting the LPS, the initial steps of invasion, which are still not completely elucidated, are mediated by the T3SS, possibly in combination with other virulence factors greatly increasing the complexity of the possible targets to be addressed or suggesting a synergistic effect of multiple antibodies and mechanisms during various steps of the infection process.

Expression and structural modifications of relevant surface antigens are probably modulated by environmental factors such as bile salts exposure, pH, temperature, glucose concentration and oxygen, which are thought to signal the bacterium its localization between the small and the large intestine. These factors need to be necessarily considered when standardizing bacterial growth/conditions to perform experiments, with deoxycholate and oxygen density combined with the presence of eukaryotic membranes are probably the most promising.

In conclusions, availability of a panel of multiple functional assays would be fundamental for the dissection of the immune response induced by *Shigella* infection or vaccination in preclinical and clinical setting, and to unravel the role played by antibodies in inducing protection against shigellosis.

## Author contributions

All authors contributed to the conception and writing of the article and approve the final version of the article.
